# Association between sleep and tinnitus in US adults: Data from the NHANES (2007–2012)

**DOI:** 10.1097/MD.0000000000040303

**Published:** 2024-10-25

**Authors:** Chao Wang, Shulin Li, Mengdi Shi, Zhu Qin, Dianyi Wang, Wentao Li, Rui Wang, Liangzhen Xie, Yan Li

**Affiliations:** a Department of Otolaryngology, The First Affiliated Hospital of Heilongjiang University of Traditional Chinese Medicine, Harbin, China; b The First Affiliated Hospital of Heilongjiang University of Traditional Chinese Medicine, Harbin, China; c Department of Pediatrics, Second Affiliated Hospital of Heilongjiang University of Traditional Chinese Medicine, Harbin, China; d Department of Otolaryngology, Cangzhou Hospital of Integrated Traditional Chinese and Western Medicine, Cangzhou, China.

**Keywords:** National Health and Nutrition Examination Survey, NHANES, sleep behaviors, sleep factors, tinnitus

## Abstract

We evaluated the relationship between sleep factors and tinnitus risk using data from the US National Health and Nutrition Examination Survey (2007–2012), focusing on adults aged 20 to 80 years. Our analysis included 4354 participants, with sleep patterns categorized as healthy, intermediate, or poor. We observed that both short (odds ratio [OR]: 1.43) and long (OR: 1.48) sleep durations increased the risk of tinnitus compared with the healthy range (7–9 hours). Additionally, sleep disturbances were significantly correlated with tinnitus (OR: 1.52), with the strongest association occurring in individuals with poor sleep patterns (OR: 1.71). The results of the weighted logistic regression analysis underscored these findings.

## 1. Introduction

Tinnitus is the perception of sound with no exterior source. However, sound can be perceived as internal or external to the head, or in one or both ears. The qualitative descriptions include buzzing, ringing, hissing, static, growling, or cicada sounds.^[1]^ The estimated prevalence of tinnitus in population surveys worldwide is 10% to 25% in people aged >18 years.^[2]^The prevalence of tinnitus is particularly high in older adults, with a 2011 survey reporting rates of up to 30.3%.^[3]^ Tinnitus-related distress can profoundly impact patient quality of life and psychological well-being, contributing to significant healthcare and socioeconomic challenges. The ongoing effects of tinnitus can lead to increased anxiety, depression, and difficulties in daily functioning, underscoring the need for effective management strategies to alleviate these burdens on individuals and the healthcare system.^[4]^ However, the treatment options for tinnitus remain poor owing to the unclear trigger mechanisms and the clinical variability of symptoms.^[5]^ Sound, tinnitus retraining, cognitive behavioral, and acupuncture therapies face problems such as insufficient evidence or difficulties in specific practices.^[6]^ Therefore, tinnitus prevention may be more effective than cure. Thus, identifying risk factors for tinnitus is essential for avoiding exposure to these factors and subsequent development of tinnitus.

The currently recognized risk factors for tinnitus include age, hearing loss, and noise.^[7]^ However, in recent years, psychological or psychosomatic symptoms including anxiety and depression are also reportedly associated with tinnitus.^[5]^ The crucial role of sleep in regulating human emotions is widely recognized. Adequate sleep is essential for maintaining emotional balance as it influences mood stability and resilience to stress. Disruptions in sleep patterns can lead to increased emotional responses, anxiety, and mood disorders, emphasizing the importance of healthy sleep habits for overall emotional well-being and the link between sleep and many adverse psychological factors such as anxiety and depression.^[8]^ In addition, clinical feedback from patients indicates that most tinnitus sufferers have varying degrees of sleep disorders. Therefore, sleep and tinnitus are likely to be directly related. A study from Japan reported sleep disturbances in 73.2% of patients with tinnitus.^[9]^ Another study from 2022 revealed that age, sleepiness in daylight hours, and psychological distress were correlated with tinnitus severity in patients with sleep difficulties.^[10]^ A meta-analysis reported that sleep disturbances were common among tinnitus patients and differed in subgroup analyses by sex and region.^[11]^ However, most previous studies focused on older adults with varying sample sizes, age groups, and definitions of sleep duration. The present study investigated the relationships between various sleep factors, including objective sleep metrics and combined sleep behaviors, and the risk of developing tinnitus in a general US adult population aged 20 to 80 years. This analysis used nationally representative data from the National Health and Nutrition Examination Survey (NHANES) to explore these associations.

The NHANES uses complex, stratified, and multistage sampling designs to assess the health of the US population. The survey protocol was approved by the National Center for Health Statistics Research Ethics Review Committee and informed consent was obtained from all participants. All procedures adhered to the established guidelines and regulations.

## 2. Methods

### 2.1. Study subjects

The NHANES included interviews and clinical examinations. The interviews consisted of questions on demographics, socioeconomics, diet, and health of the participants. The clinical examinations consist of medical, dental, and physiological measures, in addition to laboratory examinations performed by highly qualified medical experts.

In the present cross-sectional study, we analyzed publicly available data on individuals aged 20 to 80 years, comprising comprehensive and reliable information, including demographics, dietary and health-related behaviors, physical measurements, and health status, collected during the 2007–2012 waves of NHANES.

### 2.2. Sleep factors and explanation of a sleep pattern

Sleep length was evaluated using the question, “How much sleep do you typically get on a weekday or weekend night?” The responses were classified into 3 categories: short (<7 hours), normal (7–9 hours), and long (>9 hours) sleep. This classification facilitated the analysis of the relationship between sleep duration and various health outcomes.^[12]^ We defined normal sleep as a lower-risk sleep factor, and the other sleep categories as higher-risk sleep factors. Responses to the question “Have you ever informed a doctor or health professional about having sleep disturbances?” and “Have you or your doctor ever diagnosed you with a sleep disorder?” were utilized to evaluate sleep problems and sleep disorders, respectively. For the sleep behaviors described above, lower- and higher-risk sleep factors were categorized as 0 and 1, respectively, to produce an overall sleep score ranging from 0 to 3. Thus, a sleep score of 0 indicated healthy sleep patterns, while a score of 1 to 2 or 3 indicated moderate or poor sleep patterns.^[8]^

### 2.3. Assessment of tinnitus

Tinnitus was well-defined as rejoining “yes” to the question, “In the past 12 months, have you ever had ringing, roaring, or buzzing in your ears?”

### 2.4. Assessment of potential covariates

Based on previous studies, we included age, sex, race, and body mass index (BMI) as demographic factors that could influence tinnitus.^[13]^ We also included smoking, alcohol consumption, blood pressure, lipid levels, the presence of diabetes mellitus, and Patient Health Questionnaire-9 (PHQ-9) score as disease factors that may affect tinnitus as covariates. Smoking was defined as having smoked >100 cigarettes in a year, alcohol consumption was defined as having consumed >12 drinks in a year, and hypertension was assessed using a questionnaire and blood pressure measurements. The questionnaire asked, “Has your doctor ever told you that you have high blood pressure?” and “Are you currently taking medication for high blood pressure?” Additionally, hypertension was defined as a systolic blood pressure >140 mm Hg or diastolic blood pressure >90 mm Hg. Three participants were diagnosed with hypertension based on these criteria. Blood pressure measurements (systolic and diastolic) were conducted at the Mobile Examination Center.^[14]^ The National Cholesterol Education Program classifies hyperlipidemia as total cholesterol levels ≥200 mg/dL, triglyceride levels ≥150 mg/dL, high-density lipoprotein levels ≤40 mg/dL for men and ≤50 mg/dL for women, or low-density lipoprotein levels ≥130 mg/dL. Individuals who reported using cholesterol-lowering medications were also considered to have hyperlipidemia.^[15]^ The diagnostic criteria for diabetes mellitus included: a doctor’s diagnosis of diabetes, a glycohemoglobin level ≥6.5%, fasting glucose ≥7.0 mmol/L, random blood glucose level ≥11.1 mmol/L, a 2-hour oral glucose tolerance test blood glucose level of 11.1 mmol/L or higher, and use of diabetes medication or insulin. The BMI classifications were as follows: <25, 25 to 29.9, and ≥30 kg/m².^[16]^ The PHQ-9 is the most commonly used scale for diagnosing depression severity, with PHQ-9 scores of 5, 10, 15, and 20 representing mild, moderate, moderately severe, and major depressive symptoms, respectively.^[17]^

### 2.5. Statistical analysis

R Studio (version 4.2.3-2009-2023 RStudio) was used to perform the statistical analyses. The baseline tinnitus and sleep statuses were expressed as percentages. Weighted logistic regression models were used to examine the relationship between sleep factors (sleep duration, sleep difficulties, and sleep disorders) and tinnitus risk. The crude model was unadjusted, whereas model 1 was adjusted for demographic factors such as age, sex, race, and BMI. Model 2 was further adjusted for tinnitus-related adverse lifestyle factors and health conditions, including smoking, alcohol use, high blood cholesterol level, high blood pressure, diabetes mellitus, and depression severity. *P* < .05 indicates a significant difference.

## 3. Results

### 3.1. The baseline characteristics of study population

The participants’ characteristics are presented in Table 1. Among the 4354 included participants (49.69% men and 50.31% women, mean [standard deviation] age 47.47 years). According to age groups, converted to quartiles, the distribution of tinnitus differed, with a prevalence of 13.28% in the 20 to 34 years age group and 28.24%, 37.06%, and 21.42% among those aged 35 to 50, 51 to 65, and 66 to 80 years, respectively (Table 1).

**Table 1:** Characteristics of participants by tinnitus.

Variable	Total n (%)	Tinnitus		*P* value
		No n (%)	Yes n (%)	
Age (yr)	47.47 (0.73)	46.13 (0.75)	53.47 (0.82)	<.0001
Age (yr)				<.0001
20–34	1118 (25.68)	1022 (30.54)	96 (13.28)	
35–50	1093 (25.1)	920 (29.11)	173 (28.24)	
51–65	1124 (25.82)	889 (26.96)	235 (37.06)	
66–80	1019 (23.4)	763 (13.39)	256 (21.42)	
Gender				.12
Female	2137 (49.08)	1763 (51.30)	374 (45.87)	
Male	2217 (50.92)	1831 (48.70)	386 (54.13)	
Race				.002
Mexican American	426 (9.78)	352 (7.42)	74 (5.29)	
Non-Hispanic Black	1062 (24.39)	907 (11.23)	155 (8.26)	
Non-Hispanic White	1855 (42.6)	1454 (68.03)	401 (76.98)	
Other Hispanic	396 (9.1)	330 (6.14)	66 (4.03)	
Other race—including multi-racial	615 (14.12)	551 (7.18)	64 (5.44)	
Trouble sleeping				<.0001
No	3249 (74.62)	2799 (75.13)	450 (59.19)	
Yes	1105 (25.38)	795 (24.87)	310 (40.81)	
Sleep disorder				<.0001
No	3956 (90.86)	3309 (92.07)	647 (84.99)	
Yes	398 (9.14)	285 (7.93)	113 (15.01)	
Sleep duration				.01
<7	1739 (39.94)	1391 (35.76)	348 (43.96)	
7–9	2504 (57.51)	2118 (62.41)	386 (53.71)	
>9	111 (2.55)	85 (1.82)	26 (2.33)	
Smoke status				.004
No	2418 (55.54)	2065 (57.23)	353 (46.93)	
Yes	1936 (44.46)	1529 (42.77)	407 (53.07)	
Alcohol user				.33
Never	603 (13.85)	503 (9.88)	100 (8.81)	
Yes	3751 (86.15)	3091 (90.12)	660 (91.19)	
Hypertension				<.0001
No	2426 (55.72)	2114 (65.57)	312 (45.29)	
Yes	1928 (44.28)	1480 (34.43)	448 (54.71)	
DM				<.0001
No	3527 (81.01)	2961 (87.85)	566 (80.14)	
Yes	827 (18.99)	633 (12.15)	194 (19.86)	
Hyperlipidemia				<.0001
No	1344 (30.87)	1181 (32.49)	163 (19.15)	
Yes	3010 (69.13)	2413 (67.51)	597 (80.85)	
PHQ-9				<.0001
[0,4]	3339 (76.69)	2863 (81.22)	476 (64.91)	
[5,9]	622 (14.29)	480 (12.64)	142 (17.95)	
[10,14]	235 (5.4)	152 (3.77)	83 (9.57)	
[15,19]	109 (2.5)	68 (1.66)	41 (5.01)	
[20,27]	49 (1.13)	31 (0.71)	18 (2.56)	
PIR				.74
<1.38	1570 (36.06)	1253 (25.37)	317 (26.69)	
1.38–3.99	1666 (38.26)	1384 (38.71)	282 (39.24)	
≥3.99	1118 (25.68)	957 (35.92)	161 (34.07)	
BMI				.003
Normal	1328 (30.5)	1146 (31.56)	182 (24.22)	
Obese	1596 (36.66)	1249 (33.76)	347 (43.78)	
Overweight	1430 (32.84)	1199 (34.69)	231 (31.99)	
Trouble sleeping				<.001
No	3249 (72.23)	2799 (75.13)	450 (59.19)	
Yes	1105 (27.77)	795 (24.87)	310 (40.81)	
Sleep disorder				<.001
No	3956 (90.78)	3309 (92.07)	647 (84.99)	
Yes	398 (9.22)	285 (7.93)	113 (15.01)	
Sleep duration				.01
<7	1739 (37.26)	1391 (35.76)	348 (43.96)	
7–9	2504 (60.83)	2118 (62.41)	386 (53.71)	
>9	111 (1.92)	85 (1.82)	26 (2.33)	
Sleep pattern				<.0001
Healthy sleep	1990 (45.98)	1734 (48.88)	256 (32.98)	
Intermediate sleep	1578 (35.90)	1300 (35.30)	278 (38.57)	
Poor sleep	786 (18.12)	560 (15.81)	226 (28.45)	

BMI = body mass index, DM = diabetes mellitus, n = sample size, PHQ9 = Patient Health Questionnaire-9, PIR = poverty impact ratio.

Participants with tinnitus appeared to be middle-aged and older, with a higher tendency toward obesity, and were more likely to have diabetes mellitus, smoking habits, hypertension, and hyperlipidemia. The prevalence of tinnitus increased with worsening sleep pattern (Table 1).

### 3.2. Association between sleep and risk of tinnitus

**Figure 1.**
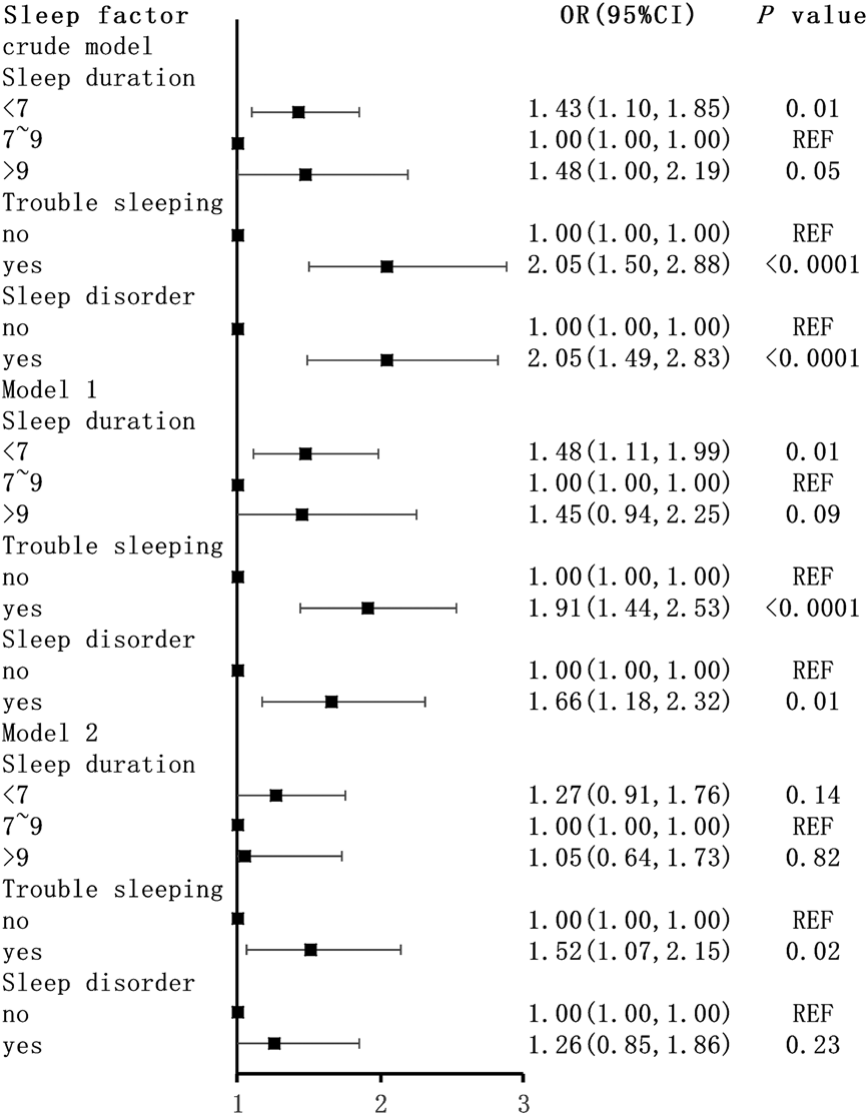
Logistic regression analyses of the association between sleep factors and Tinnitus. The data are presented as ORs, 95% CIs, and *P* values. Crude model: unadjusted. Model 1: adjusted for sex, race, age, smoke, alcohol user, BMI, and PIR. Model 2: adjusted for model 1 and hypertension, hyperlipidemia, PHQ-9, and DM. BMI = body mass index, CIs = confidence intervals, DM = diabetes mellitus, ORs = odds ratios, PHQ-9 = Patient Health Questionnaire-9, PIR = poverty impact ratio.

In the unadjusted (crude) model, participants who slept <7 hours or >9 hours were more likely to develop tinnitus (odds ratio [OR]: 1.43, 95% confidence interval [CI]: 1.10–1.85 and OR: 1.48, 95% CI: 1.00–2.19, respectively; Fig. 1). Additionally, participants with sleep disturbances and disorders were also more likely to develop tinnitus (OR: 2.05, 95% CI: 1.50–2.88 and OR: 2.05, 95% CI: 1.49–2.83, respectively). After adjusting for potential confounders in model 2, short (OR: 1.27, 95% CI: 0.91–1.76) and long (OR: 1.05, 95% CI: 0.64–1.73) sleep durations were not significantly associated with tinnitus. However, sleep disturbances and disorders continued to reveal a significant relationship with tinnitus (OR: 1.52, 95% CI: 1.07–2.15; OR: 1.26, 95% CI: 0.85–1.86; Fig. 1).

**Figure 2.**
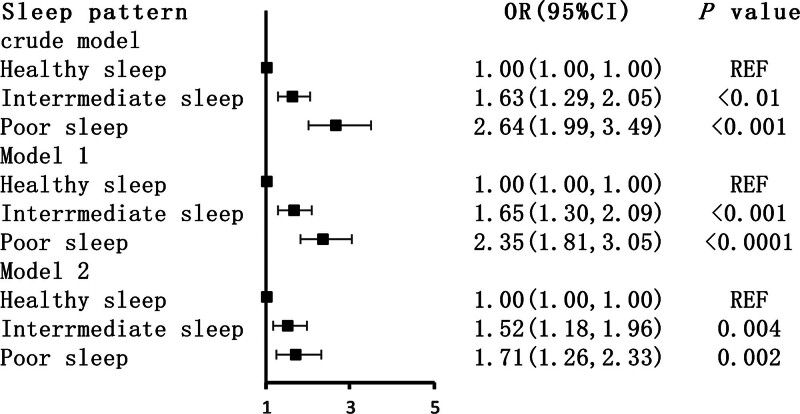
Logistic regression analyses of the association between sleep patterns and tinnitus. Crude model: unadjusted. Model 1: adjusted for sex, race, age, smoke, alcohol user, BMI, and PIR. Model 2: adjusted for model 1 and hypertension, hyperlipidemia, PHQ-9, and DM. BMI = body mass index, CIs = confidence intervals, DM = diabetes mellitus, ORs = odds ratios, PHQ-9 = Patient Health Questionnaire-9, PIR = poverty impact ratio.

The relationships between sleep patterns (sleep duration, sleep difficulties, and sleep disorders) and tinnitus are presented in Figure 2. In the crude model, participants with poor sleep patterns were more likely to develop tinnitus compared with those with healthy sleep patterns (OR: 2.64, 95% CI: 1.99–3.49). After adjusting for potential confounders (model 2), this relationship remained significant (OR: 1.71, 95% CI: 1.26–2.33).

### 3.3. The association of sleep patterns with tinnitus risk by ages

**Figure 3.**
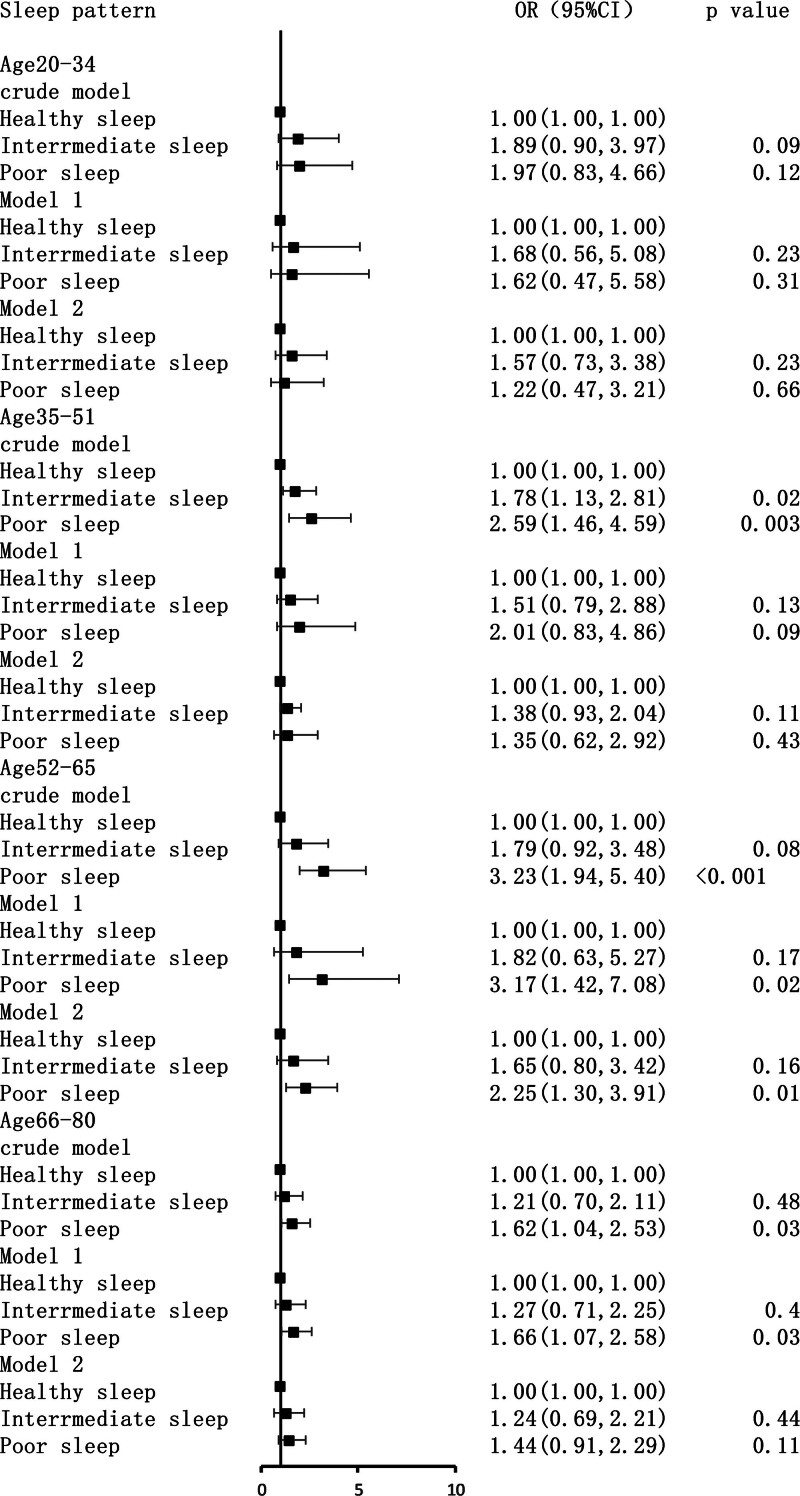
Logistic regression analyses of the association between sleep patterns and tinnitus stratified by age. The data are presented as ORs, 95% CIs, and *P* values. Crude model: unadjusted. Model 1: adjusted for sex, race, smoke, alcohol user, BMI, and PIR. Model 2: adjusted for model 1 and hypertension, hyperlipidemia, PHQ-9, and DM. BMI = body mass index, CIs = confidence intervals, DM = diabetes mellitus, ORs = odds ratios, PHQ-9 = Patient Health Questionnaire-9, PIR = poverty impact ratio.

After stratification by age, the association between poor sleep patterns and increased tinnitus remained significant across all age groups, particularly among those aged 50 to 65 years. In contrast, the trend was less pronounced in the 66- to 80-year age group (Fig. 3).

### 3.4. The association of sleep patterns with tinnitus risk by sex

**Figure 4.**
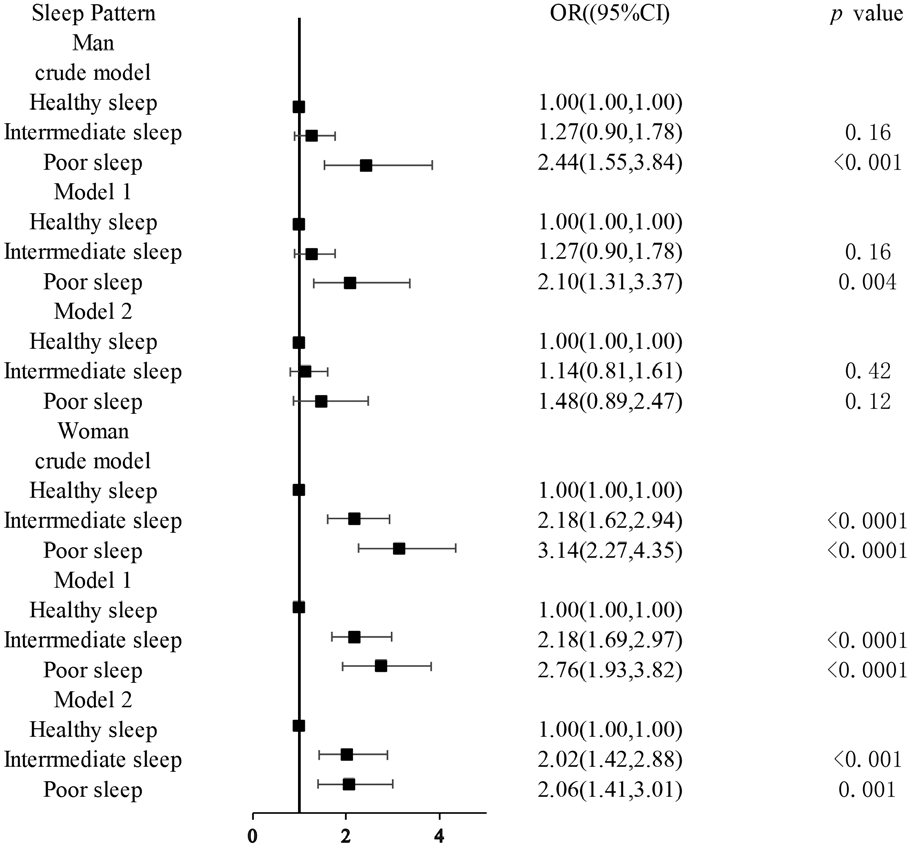
Logistic regression analyses of the association between sleep patterns and tinnitus stratified by sex. Crude model: unadjusted. Model 1: adjusted for race, age, smoke, alcohol user, BMI, and PIR. Model 2: adjusted for model 1 and hypertension, hyperlipidemia, PHQ-9, and DM. BMI = body mass index, CIs = confidence intervals, DM = diabetes mellitus, ORs = odds ratios, PHQ-9 = Patient Health Questionnaire-9, PIR = poverty impact ratio.

After sex stratification, the increasing trend of the association of tinnitus with poor sleep patterns remained significant among women (Fig. 4). After adjusting for potential confounders (model 2), this relationship remained significant (OR: 2.06, 95% CI: 1.41–3.41).

### 3.5. Sleep as a mediator in the associations between tinnitus and depression

As depression is associated with tinnitus and sleep with depression, sleep may be the intermediary between depression and tinnitus. Therefore, we assessed whether sleep was an intermediary between depression and tinnitus. The results of the mediation analysis demonstrated that sleep was not a mediator between these factors (Table 2).

**Table 2:** Sleep as a mediator in the associations between tinnitus and depression.

Mediation effect	Estimate	95% CI lower	95% CI upper	*P* value
Total effect	0.232	0.293	0.27	<.001
Mediation effect	0.000	0.000	0.000	1
Direct effect	0.232	0.193	0.27	<.001
Proportion mediated	0.000	0.000	0.000	1

CI = confidence interval.

## 4. Discussion

Tinnitus is a phantom sound that affects patient quality of life; thus, effective treatments are urgently needed. However, the etiologies of tinnitus remain unknown and methods for assessing and measuring tinnitus are being developed. Among the reported risk factors and comorbidities for tinnitus, sleep deprivation should not be ignored, as sufficient rest helps maintain body and emotional functions. Previous studies reported that up to 76% of patients with tinnitus experience insomnia.^[18]^

This is the first investigation of the relationship between sleep patterns and tinnitus in a large, nationally representative study. Our findings demonstrated the association of sleep problems (sleep disturbances and sleep disorders) with tinnitus. Our results also revealed the association between sleep duration, difficulties, disorders, and the risk of tinnitus, with participants with poor sleep patterns having a higher risk of developing tinnitus. Among the 4354 included participants, 49.08% of women and 50.92% of men reported tinnitus. The difference between the 2 groups was not large and was comparable to that reported previously.^[19]^ The prevalence might also be affected by individual variations in susceptibility and environmental factors. Sleep quantity and quality are fundamental components of restorative sleep and a causal relationship exists among depression, sleep, and tinnitus. Therefore, it is reasonable to hypothesize that the relationship between tinnitus and sleep is similar to that between depression and sleep. A longitudinal study demonstrated that symptoms or biomarkers of insomnia, as assessed using polysomnography, can increase the prevalence of depression by 2.2- to 5.3-fold.^[20]^ In the present study, sleep difficulties were associated with tinnitus in the fully adjusted model (OR: 1.52, 95% CI: 1.07–2.15). This result is consistent with the findings of a previous study that concluded that sleep disturbances are more common in patients with tinnitus. Another study reported that patients with sleep difficulties experienced more severe tinnitus than those without.^[21]^ Moreover, patients with tinnitus have a longer sleep latency.^[22]^ This circumstance may additionally be associated with fluctuating serotonin levels, which are associated with sleep regulation. Patients with tinnitus show a reduced volume of the subcallosal region, a portion of which partially overlaps with the locus coeruleus. Consequently, corresponding atrophy may be observed in the nucleus accumbens of these individuals. Moreover, the locus coeruleus is the primary source of serotonergic neurons; thus, this reduced volume may lead to sleep disturbance in patients with tinnitus.^[23]^

Localized cortical arousal is typical of heteromorphic sleep.^[24]^ Sleep disorders caused by local cortical arousal include night terrors and sleepwalking, which affect sleep patterns and, therefore, tinnitus. Night terrors are positively correlated with chronic tinnitus.^[25]^ Patients with tinnitus and anxiety exhibit activation of the intrinsic network and limbic structures.^[26]^ An example is neuronal hyperactivity in the nucleus ambiguus. Although the persistence of this abnormal activity during sleep has not been studied, it may contribute to variations in normal cognitive functioning, similar to dissociative states. A recent study reported a high incidence of heteromorphic sleep and sleep disorders in patients with tinnitus.^[27]^ Another perspective on the interaction between tinnitus and cognitive function can be illustrated through sensory stimuli, whose perception is akin to subjective hallucinations. Historically, sleep has been described as a state with relative sensory and typical disconnections.^[28]^ However, cognitive function remains relatively receptive to auditory stimuli during sleep. Auditory evoked potentials are typically preserved at the level of the auditory cortex, and persistent tinnitus can significantly affect sleep quality.^[29]^ Moreover, increasing evidence indicates that tinnitus is a whole-brain phenomenon, involving ongoing changes in brain activity across the frontal, parietal, and limbic regions—areas directly linked to sleep regulation and expression. These findings highlight the widespread vulnerability of sleep to tinnitus-related disorders.^[29]^ At the same time, a growing body of research demonstrates that tinnitus is a whole-brain phenomenon, involving ongoing changes in brain activity across the frontal, parietal, and limbic regions—areas directly linked to sleep regulation and expression. This highlights the widespread vulnerability of sleep to tinnitus-related disturbances.^[25]^

Our study used a large dataset from a nationally representative population sample. The NHANES sampling methodology ensured that the sample was randomly selected and representative of the entire US population. However, our study has several limitations. First, as this was a cross-sectional study, we cannot rule out reverse causality owing to the study design. Second, the types of sleep problems were not clearly defined in this study. Finally, all sleep-related data were self-reported, which introduced potential recall bias and lacked objectivity compared with sleep-monitoring methods.

## 5. Conclusions

Overall, the results of this study emphasized the complex and nuanced relationship between sleep-related issues and tinnitus risk. Further research should explore the causal or bidirectional relationships between sleep disorders and tinnitus. In addition, investigating the genetic associations and underlying mechanisms between these sleep disorders and tinnitus is essential for developing effective prevention and management strategies.

## Acknowledgments

Thanks to Zhang Jing (Second Department of Infectious Disease, Shanghai Fifth People’s Hospital, Fudan University) for his work on the NHANES database. His outstanding work, nhanesR package and webpage, makes it easier for us to explore NHANES database.

## Author contributions

**Writing – original draft:** Chao Wang, Mengdi Shi.

**Writing – review & editing:** Chao Wang, Yan Li, Shulin Li.

**Data curation:** Zhu Qin.

**Methodology:** Dianyi Wang, Rui Wang.

**Project administration:** Wentao Li.

**Conceptualization:** Liangzhen Xie.
